# Nursing Assistant Training in VHA Community Living Centers: Resources and Recommendations

**DOI:** 10.1093/geroni/igaf122.764

**Published:** 2025-12-31

**Authors:** Katherine Kennedy, Nathalie Mcintosh, Sylvia Haigh, David Mohr, Ciaran Phibbs, Whitney Mills

**Affiliations:** Providence VA Healthcare System, Providence, Rhode Island, United States; THRIVE, Providence VA Medical Center, Providence, Rhode Island, United States; VA Providence Healthcare System, Providence, Rhode Island, United States; Veterans Health Administration, Mason, Ohio, United States; Health Economics Resource Center, VA Palo Alto Health Care System, Menlo Park, California, United States; Brown University, Providence, Rhode Island, United States

## Abstract

Quality training is one component of direct care job quality in long-term care based on non-VA research. This study aimed to identify patterns in nursing assistant (NA) training in Veterans Health Administration Community Living Centers (CLCs; nursing homes). We recruited 2 high-performing and 2 low-performing CLCs based on NA intent to stay from a 2022 survey and completed 6-9 interviews at each (N = 30). We employed qualitative content analysis based on Paraprofessional Healthcare Institute’s (PHI) Five Pillars of Direct Care Job Quality to understand training factors impacting intent to stay. The high-performing CLCs (88% average intent to stay) provided structured and well-coordinated orientation, longer floor orientation, offered NA skills workshops, and leveraged existing relationships with clinical resource nurses (CRNs) who supplemented nurse manager coaching. Whereas the low-performing CLCs (32% average intent to stay) had less well coordinated orientations, shorter floor orientation, lacked regular skills workshops, experienced turnover in nurse educator/CRNs, and reported NA training as an area needing improvement. Recommendations included: NA-tailored, standardized training in various topics (i.e., mental health, dementia, end-of-life, nursing) and more continuing education; synergizing NA and nurse educator/CRN relationships and directly communicating with NAs about training opportunities; and modifications needed to address barriers related to time, staffing, computer access and learning preferences. This study confirms the importance of training to NA job satisfaction in VHA CLCs. Ways to improve training and access to training (e.g., microlearning) may improve job satisfaction and retention.

